# Exploring reasons for recruitment failure in clinical trials: a qualitative study with clinical trial stakeholders in Switzerland, Germany, and Canada

**DOI:** 10.1186/s13063-021-05818-0

**Published:** 2021-11-25

**Authors:** Matthias Briel, Bernice S. Elger, Stuart McLennan, Stefan Schandelmaier, Erik von Elm, Priya Satalkar

**Affiliations:** 1 Basel Institute for Clinical Epidemiology and Biostatistics, Department of Clinical Research, University of Basel and University Hospital Basel, Spitalstrasse 12, 4031 Basel, Switzerland; 2 Department of Health Research Methods, Evidence, and Impact, McMaster University, Hamilton, Ontario Canada; 3 Institute for Biomedical Ethics, University of Basel, Basel, Switzerland; 4 Center for Legal Medicine, Unit for Health Law and Humanitarian Medicine, Faculty of Medicine, University of Geneva, Geneva, Switzerland; 5 Institute of History and Ethics in Medicine, TUM School of Medicine, Technical University of Munich, Munich, Germany; 6 Cochrane Switzerland, Centre for Primary Care and Public Health (Unisanté), University of Lausanne, Lausanne, Switzerland

**Keywords:** Randomized clinical trials, Interview study, Poor recruitment, Reasons for recruitment failure, Qualitative analysis

## Abstract

**Background:**

Poor participant recruitment is the most frequent reason for premature discontinuation of randomized clinical trials (RCTs), particularly if they are investigator-initiated. The aims of this qualitative study were to investigate (1) the views of clinical trial stakeholders from three different countries regarding reasons for recruitment failure in RCTs and (2) how these compare and contrast with the causes identified in a previous systematic review of RCT publications.

**Methods:**

From August 2015 to November 2016, we conducted 49 semi-structured interviews with a purposive sample of clinical trial stakeholders. This included investigators based in Germany (*n* = 9), Switzerland (*n* = 6) and Canada (*n* = 1) with personal experience of a discontinued RCT and 33 other stakeholders (e.g., representatives of ethics committees, clinical trial units, pharmaceutical industry) in Switzerland. Individual semi-structured qualitative interviews were conducted and analyzed using thematic analysis.

**Results:**

Interviewees identified a total of 29 different reasons for recruitment failure. Overoptimistic recruitment estimates, too narrow eligibility criteria, lack of engagement of recruiters/trial team, lack of competence/training/experience of recruiters, insufficient initial funding, and high burden for trial participants were mentioned most frequently. The interview findings largely confirm the previous systematic review on published reasons for recruitment failure. However, eight new reasons for recruitment failure were identified in the interviews, which led to the checklist of reasons for recruitment failure being revised and a new category describing research environment-related factors being added.

**Conclusions:**

This study highlights the diversity of often interlinked reasons for recruitment failure in RCTs. Integrating the findings of this interview study with a previous systematic review of RCT publications led to a comprehensive, structured checklist of empirically-informed reasons for recruitment failure. The checklist may be useful to guide further research on interventions to improve participant recruitment in RCTs and helpful for trial investigators, research ethics committees, and funding agencies when assessing trial feasibility with respect to recruitment.

**Supplementary Information:**

The online version contains supplementary material available at 10.1186/s13063-021-05818-0.

## Background

Participant recruitment is a crucial step in the planning and conduct of randomized clinical trials (RCTs), and poor participant recruitment is the most frequently identified reason for premature discontinuation of RCTs [1, 2]. This is particularly problematic in investigator-sponsored RCTs as opposed to industry-sponsored trials, because of scarce human and financial resources, often underdeveloped research networks, overoptimistic recruitment estimates, and insufficient planning [3, 4]. When RCTs are prematurely discontinued due to poor recruitment of participants, the respective research questions typically remain unanswered wasting limited resources and efforts by investigators, trial staff, and already enrolled participants [5]. Furthermore, premature RCT discontinuation is a strong risk factor for non-publication [1]. Failure to share data from discontinued RCTs (e.g., through journal publication, study registry, or trial website) means that existing data cannot contribute to systematic reviews and meta-analyses, and failure to share lessons learnt puts future RCTs at risk of repeating potential mistakes in planning and conduct [1, 4].

Several qualitative and quantitative studies have already suggested a large number of barriers and facilitators for participant recruitment in RCTs, but these typically focused on specific countries, disciplines, or settings [3, 6–12]. A comprehensive and generic summary of causes for recruitment problems leading to trial discontinuation could be useful for trial investigators to be aware of potential risk factors and to allow for preventive action according to the specific context of each individual trial. In a previous systematic review, we collected reasons of recruitment failure from RCT reports from any discipline, country, or setting that were published between 2010 and 2014 and drafted a checklist for trial investigators and other stakeholders [4]. In that previous systematic review, we identified 28 different reasons for recruitment failure reported by trial investigators, with overoptimistic recruitment estimates in conjunction with narrow eligibility criteria being the most commonly reported reasons. It is unclear, however, if there are other relevant reasons for recruitment failure in RCTs that are not currently reported in the literature, and that could complement the existing checklist. We therefore decided to conduct a qualitative interview study on the causes for recruitment failure in RCTs with a broad spectrum of trial stakeholders. We have previously reported Swiss-specific reasons for recruitment failure [3]; however, the aims of the present study were to examine (1) the views of trial investigators from three different countries and other stakeholders (e.g., representatives of ethics committees, clinical trial units, pharmaceutical industry) from Switzerland regarding perceived root causes for recruitment failure in RCTs not specific for a country, discipline, or setting and (2) how these compare and contrast with the causes identified in the previous systematic review of RCT reports [4].

## Methods

### Context and study population

This qualitative study comprised in-depth interviews with RCT stakeholders. We used the database of a prior quantitative study on early discontinuation of clinical trials to identify trial investigators with experience of recruitment failure [1]. The prior study included RCT protocols approved by one of six research ethics committees from Switzerland, Germany, or Canada. The collaboration with ethics committees in the three countries was established through the professional network of MB. The three high-income countries have similar trial infrastructure, funding mechanisms, clinical research work force, training opportunities, regulatory norms, and a similarly high prevalence of recruitment failure [1]. From these trial investigators with experience of trial discontinuation due to poor participant recruitment, we were keen to understand in detail the circumstances in which their trial was stopped and their reflections on various factors that led to poor recruitment and eventual discontinuation.

Through our professional networks in Switzerland, we identified and approached diverse categories of other RCT stakeholders who could elaborate on reasons of trial discontinuation due to poor recruitment in academic research settings based on their experience in planning, implementing, evaluating, or regulating clinical trials. These included representatives of ethics committees, clinical trial units, international pharmaceutical companies, patient organizations (i.e., patients with lived experience of participating in a clinical trial), a funding agency, public health authority, drug regulatory authority, cancer research network, and clinicians (medical doctors) with experience in clinical trials (Table 1).

**Table 1:** Description of interviewees

Description	*N*
**Total number of persons interviewed**	**49**
*Trial investigators with experience of recruitment failure in a clinical trial identified in previous* research *[*1*]*	**16**
• From Germany	9
• From Switzerland	6
• From Canada	1
*Other clinical trial stakeholders from Switzerland*	**33**
• Representatives of international pharmaceutical companies	10
• Clinicians (medical doctors) with experience in clinical trials	8
• Representatives of clinical trial units	6
• Representatives of ethics committees	3
• Representatives of patient organizations (people with lived experience of being a patient and participating in a clinical trial)	2
• Representative of funding agency	1
• Representative of public health authority	1
• Representative of drug regulatory authorities	1
• Representative of cancer research network	1

All identified potential interviewees could be directly contacted via email which described in detail the goal of the study, process of ensuring confidentiality, and privacy across the research life cycle including eventual publications and what they could expect in terms of time commitment if they would agree to take part in this study.

### Data collection and processing

With a goal of obtaining comprehensive insights on the topic of trial discontinuation, we used a purposive sampling strategy with inclusion of at least one representative for each of the listed stakeholder groups to allow for a broad range of perspectives. A semi-structured interview guide (Appendix A) was developed, informed by insights from published articles, which we identified in a Medline search at the time. The interview guide included topics such as the causes of insufficient recruitment and trial discontinuation, possible methods to prevent insufficient recruitment, and implications of trial discontinuation for science, clinical practice, researchers, and trial participants. The interview guide was discussed within the research group and pilot tested with two stakeholders. No significant adaptations of the interview guide were necessary. We eventually included the two pilot interviews in the final data set as they were held under the same conditions and included the same content as subsequent interviews.

In line with interviewees’ preference, interviews were conducted in English, in person or through phone or a Skype call by PS (a female, mid-career researcher with expertise in qualitative research methods and empirical bioethics who conducted 24 interviews) or by MB (a male, senior clinical trial professional and methodologist who carried out the remaining 25 interviews). Although all interviewees had received an email with study information and invitation to participate in our study, we repeated key aspects of this information at the beginning of each interview and obtained permission to record the conversation on an audio-device and oral informed consent. All interviewees allowed us to record the conversation on an audio device. During the interviews, we were sensitive in our approach especially when we invited trialists to reflect on their discontinued trials and encouraged them to describe all events and reasons that ultimately led to trial discontinuation. The semi-structured interview guide facilitated the conversation between the researchers and the interviewees where interviewees had ample opportunity to bring up the experiences/arguments that they deemed relevant to the topic under investigation. It also helped the researchers to keep the conversation focused on the topic of enquiry without needing to follow a rigid structure. In addition to open-ended questions, we also used probing questions to gain clarity on the views of the interviewees and to generate discussion. The mode of interview (phone versus in person) and the characteristics of the interviewers such as gender, seniority, primary professional identity, or topic expertise did not appear to influence the data quality (e.g., the interview duration, the depth and breadth of insights generated through each interview). Depending on other commitments of interviewees, their time constraints and interest in the topic, the interview duration ranged between 10 and 60 min, the median being 35 min.

Research assistants who had signed a confidentiality agreement transcribed all interviews verbatim and removed all personal identifiers such as name, professional profile, and affiliation. PS verified all transcripts against original recordings to ensure content accuracy. Although transcripts were returned to all interviewees, only three reviewed their interview transcript, adding a few clarifications or suggesting some syntax modifications but did not ask to remove any sections.

### Data analysis

We analyzed the data simultaneously (e.g., listening to audio recordings of interviews conducted by each interviewer, discussing initial impressions and assessing the need to modify our interview guide in light of any new topics brought up by interviewees during the conversation) while continuing data collection. PS and MB read the transcripts several times to gain familiarity and data immersion. PS used a qualitative data analysis software MAXQDA licensed by the University of Basel to code and organize all the data whereas MB manually coded few interviews. Both performed inductive coding according to thematic analysis [13]. In initial discussions, PS and MB compared and discussed their individual coding and agreed on a coding structure that PS followed for all the interviews. The codes and resulting themes generated through an iterative process were discussed in several meetings with the entire research team until differences in interpretation were resolved and consensus was reached.

After completing the thematic analysis, we carried out root cause analysis of factors that led to trial discontinuation [14, 15]. Our aim was to compare the reasons for poor recruitment and mechanisms of discontinuation gathered through interviews with those previously published in a qualitative systematic review of discontinued RCTs [4]. We expected that the insights from interviews will provide more nuance than those reported in published RCTs. Our first step was to collect all sections of transcripts where reasons for trial discontinuation were discussed, several of which provided rich contextual details. MB and PS challenged each reason, experience, or explanation provided by the interviewee with a series of “why” questions, each answer forming the basis of the next “why” question. This allowed identification of the main underlying cause that led to trial discontinuation. These root causes were compiled according to their frequency and grouped together. We then modified and expanded our previous classification of reasons (checklist) by adding details to factors already reported and re-structured categories of reasons leading to trial discontinuation. The process of synthesizing insights on root causes for trial discontinuation from the interview study and generating themes was highly iterative and required multiple rounds of discussion among the authors. Through the subsequent process of completing the thematic analysis before starting the root cause analysis and comparison with previously reported reasons of trial discontinuation due to poor recruitment (the checklist), we minimized the influence of reasons reported in the earlier published checklist on our inductive data coding and analysis. MB was the lead author of the research team that had created the earlier checklist; however, PS was not involved in that earlier study and could bring in a new perspective.

We analyzed and discussed the themes and root cause analysis with several iterative rounds of discussion within the research team. We verified and triangulated our qualitative findings with published literature and discussed unexpected results with experts in the field. Our multidisciplinary research team (medicine, public health, epidemiology, medical anthropology, and medical ethics) facilitated rigorous data analysis and minimized biased interpretation. MB, PS, EvE, StS, and BE are trained in medicine. BE, SM, and PS had additional training in research ethics and MB and EvE in public health. MB, StS, and EvE brought significant experience and familiarity with the clinical research enterprise into this research project and PS, SM, and BE contributed experience in qualitative research. If one of the interviewers was too closely associated with an interviewee, the other interviewer conducted that interview to minimize bias arising from familiarity with the interviewee and hence accepting certain aspects of their views without further questions. It also helped in neutralizing the dynamics between the researcher and the interviewee that could arise from prior or ongoing professional relationships. We monitored the influence of interviewer characters on quality of data generated (e.g., richness of the data, interview duration, the breadth and depth of the conversations). We report the results according to the Standards for Reporting Qualitative Research (SRQR, http://www.equator-network.org/reporting-guidelines/srqr/, Appendix B).

### Ethical approval and data sharing

The study was exempted from the ethics review by the regional ethics committee of Northwestern and Central Switzerland according to the Swiss Human Research act (EKNZ UBE-15/50). The Swiss Human Research Act is applicable if personal health data or biological samples are collected from study participants or if the study includes a vulnerable population. The interviewees in this qualitative study were not considered as a vulnerable population and the topic under investigation was not deemed highly sensitive or potentially stigmatizing for interviewees. With strict pseudonymization and confidentiality policy, risk of professional harm to interviewees was excluded or was at most minimal. Since we did not seek their permission to make the interview data (transcripts) available on a data-sharing platform, the original data on which this manuscript is based cannot be made publicly available, but will be shared on a case-by-case basis.

## Results

### Profile of interviewees

Of the 114 invited trial investigators and other stakeholders, 49 (43%) agreed to participate and were interviewed by MB or PS between August 2015 and November 2016. The interviewees included 16 trial investigators based in Germany (*n* = 9), Switzerland (*n* = 6), and Canada (*n* = 1) with personal experience of a discontinued RCT, and 33 other stakeholders in Switzerland. Their characters are described in Table 1. Several interviewees provided multiple perspectives on trial discontinuation depending on their current or former professional role. For example, some of the industry representatives had been academic investigators in their early professional life and could retrospectively reflect on challenges of conducting investigator-initiated trials in comparison to their current role in planning and monitoring large multi-center industry-sponsored RCTs.

### Reasons for recruitment failure reported in interviews

We identified a total of 29 reasons for recruitment failure from the root cause analysis of insights provided by the interviewees during in-depth interviews, which we grouped under five main categories: (1) funding-related, (2) research environment-related, (3) design-related, (4) trial team/recruiter-related, and (5) participant-related (Table 2).

1. Funding-related factors

**Table 2:** Reasons for recruitment failure obtained from interviews and journal publications

Reported reasons	Frequency (from interviews ***n*** = 49) ^**a**^	Frequency (from publications ***n*** = 131) ^**g**^ [4]
**Funding-related**		
Initial funding insufficient (including lack of funding for planning; inadequate financial planning)	10	15
Additional funding for recruitment escalation/prolongation unavailable	1	6
*Initial funding withdrawn when slow recruitment became apparent*	*0*	*4*
**Research environment-related**		
Delay in opening recruitment sites (e.g., delayed ethical approval, new regulatory acts)	8	7
High bureaucratic burden for clinical researchers (e.g., GCP regulations)	2	3
**Patients not seeking trials in high-quality health care system with many options (lack of incentive)**	**6**	**0**
**Decentralized health care system with many small hospitals and few referrals in Switzerland (e.g.**, **because of restrictive regional insurance contracts; in Germany peripheral hospitals often provide specialized care and refer only severely ill patients to tertiary care hospitals)**	**4**	**0**
General mistrust in research (unbalanced views in media, no public figures as role model to encourage trial participation)	3	4
Concurrent competing trials (particularly if other trial in same patient population pays more—see below “Financial conflicts of interest”)	6	11
New evidence from other study about effectiveness of trial intervention (e.g., change of standard care during prolonged trial)—affects “equipoise” for recruiters and participants (see below)	4	28
**Design-related** ^**b**^		
**Research question insufficiently compelling (e.g.**, **perceived as little relevant for patients and the scientific community)**	**5**	**0**
Low prevalence of condition of interest (seasonal effects, rare diseases)	7	2
Too narrow eligibility criteria	12	59
Overoptimistic/unreliable recruitment estimates ^c^ (e.g., no pilot study; no empirical data; insufficient feasibility checking; weak commitment from centers)	26	10
**Too few recruiting sites planned or too few study staff (e.g.**, **recruiters, limited engagement of study nurses)**	**7**	**0**
Recruitment insufficiently compatible with routine clinical practice (e.g., urgent transfers from intensive care, different treatment availabilities at different centers or at weekends, referrals too late to tertiary care) ^d^	7	11
Lack of methodological/logistical support (e.g., from contract research organization, or clinical trial unit)	4	7
Trial design too difficult to explain or implement (e.g., complex interventions, factorial design)	7	4
*Unclear eligibility criteria or enrolment process (e.g.*, *regarding timing of randomization or responsibilities of involved investigators/personnel)*	*0*	*6*
*Ineffective screening/advertising strategy (e.g.*, *email instead of phone call, newspaper campaign only)*	*0*	*5*
*Patients approached in inconvenient situation (e.g.*, *women in labor, patients in ICU)*	*0*	*5*
**Lack of patient engagement in trial design/planning**	**4**	**0**
**Trial team/recruiter-related**		
Lack of equipoise ^e^	4	34
High administrative burden/time constraints/other priorities	4	11
Lack of incentive (e.g., financial, academic recognition, career advancement)	2	4
Lack of engagement/cooperation (e.g., recruiters (practicing clinicians) unaware of trial, recruiters not involved with study team, departments not referring patients to trial recruiters)	12	4
**Lack of competence/training/experience (includes inadequate planning; “enthusiasm is not enough,” staff turnover)**	**11**	**0**
**“Trial fatigue” (motivation compromised due to recurrent prolongation of recruitment period)**	**3**	**0**
Financial conflict of interest (e.g., trial results favoring conservative treatment over surgery may lead to less earnings; other trial in same patient population pays more—see above “Concurrent competing trials”)	2	1
**Participant-related**		
Lack of equipoise ^f^	6	40
High burden (e.g., many visits, invasive procedure/biopsies, long questionnaires, costs)	9	20
Language or cultural barriers	1	4
*Lack of financial incentive*	*0*	*2*
*Lack of encouragement from patient support organizations*	*0*	*1*
**Lack of trust due to short -term relationship with healthcare team (e.g.**, **acute care vs chronic conditions (e.g.**, **dialysis patients))**	**2**	**0**

Reasons identified in interviews only are marked in bold and those identified in published reports only are marked in cursory font

^a^We obtained 179 accounts of explanations for poor participant recruitment from 49 qualitative interviews. These explanations could be classified into 29 reasons. If a respondent described more than one possible factor (explained in parenthesis) that contributed to the same reason, it was counted only once

^b^Need for simpler/more pragmatic design

^c^Reasons for “overoptimism”: intention to convince reviewers of trial feasibility in case of a grant proposal/ethics review or when applying for participation in an industry trial

^d^Requirement of intensified cooperation/communication among research team or intensified training of recruiters

^e^Equipoise means the uncertainty about benefits and harms of an intervention or about the superiority of one intervention over another, and is a pre-requisite for randomization. Lack of equipoise was reported as concerns about disadvantages for participants, vulnerable populations (e.g., children), trial questioning current practice, loss of professional autonomy, dislike of randomization to unwanted intervention, worry about doctor-patient relationship, recommendations from opinion leaders, media information favoring a particular treatment, and inadvertent use of unbalanced terminology such as gold standard

^f^Equipoise means the uncertainty about benefits and harms of an intervention or about the superiority of one intervention over another, and is a pre-requisite for randomization. Lack of equipoise was reported as influenced by next of kin or caring physician, concerns regarding side effects or potential diagnosis, vulnerable populations (e.g., children), concerns regarding randomization to unwanted intervention, unwillingness to receive placebo or no treatment, loss of personal autonomy/reluctance to become a “guinea pig,” worry about doctor-patient relationship, and media information favoring a particular treatment

^g^Most publications mentioned more than one reason

Interviewees repeatedly stated that securing sufficient funding for the entire duration of a RCT was one of the biggest challenges for investigator-initiated trials (see Table 3 for illustrative quotes). This includes mistakes of principal investigators (PIs) while planning the RCT budget, insufficient funding for trial feasibility assessments, an optimistic trial start with inadequate funding for the whole trial, and “inability of the PI to procure additional funds during the course of the trial.” Recruitment of trial participants often takes longer than anticipated, but budgets for investigator-initiated trials are typically tight without a buffer for extended trial duration. When PIs fail to secure additional funds, trials are often prematurely discontinued. Some interviewees elaborated how limited overall funding available for clinical trials adversely influences manpower planning which in turn slows down participant recruitment as elaborated in the quote below.

“If you have a limited amount of centers and you cannot finance the staff needed, you either can - if you are in the position in a hospital to tell your normal staff to include patients which are not on a study budget but which are on the hospital budget. Or else - if you cannot do that, then you just have to reduce the research staff. For example, instead of recruiting 24 hours a day, you can only for office hours, which again gives a selection bias.” R3 Clinical trialist

**Table 3:** Quotations illustrating funding-related factors

The second problem, I guess, is the amount of funding. If you compare the amount of money investigator initiated trials are funded with as compared to industry initiated trials, there is a big discrepancy with the factor of I guess about ten, although both trials have the same - now - the same bureaucratic machinery to deal with. So this is certainly one risk that - I mean you apply for a fund for a certain budget, say half a million, one million. Then you get funded with one third of it, which then often makes it more difficult to actually complete the trial with a number anticipated which then leads to underpowerment. **R3 Clinical trialist** I think another important consideration obviously is always a financial or appropriate staff. So usually physicians are overenthusiastic in doing trials but sometimes they don’t allocate enough human resources in order to do the patient work, the administrative work. And so underfunding is certainly also - plays a role in impeding patient recruitment. **R21 Clinical trialist** No. Seriously, we just don’t have enough money. There is very, very little money with XXXX grant. **T1 Trial coordinator of a discontinued trial**	

Several interviewees mentioned that merely increasing publicly available funds for investigator-initiated trials is insufficient unless funding agencies also conduct rigorous feasibility assessment of trial protocols. A clinical trial unit representative argued that without proof of feasibility and a clear recruitment plan based on empirical participant data, a funding body should not fund RCTs to ensure judicious utilization of limited resources.

2. Research environment-related factors

A majority of our interviewees reflected on changes in the regulatory environment for clinical trials over the course of their professional lives, sometimes spanning over a few decades. They argued that getting a study site ready for recruitment in the current research environment involves much more stringent regulatory and ethical oversight and bureaucratic procedures than before (see Table 4 for illustrative quotes). Almost none of them criticized the regulatory measures; they valued those checks and controls put in place to ensure trial participant safety. However, they believed that this could cause significant delay in initiating recruitment if the time needed to get all the approvals was not accounted for in the planning phase.

**Table 4:** Quotations illustrating research environment-related factors

I think a second consideration in trials is certainly also regulatory. So for example, I tell you one important issue of recruitment relates to women. And we are always discussing, why is it so difficult to recruit women. But one really daily hurdle to overcome is the pregnancy test. So obviously this comes out of an era where there has been drug adverse effects, but it has been carried on into more recent trials. And it becomes ridiculous to the degree that we are, for example, studying patients who are octogenarians, yet if we include female patients we have to prove that they have a negative pregnancy test. And that shows you a little bit that also regulatory hurdles can play a certain role in impeding recruitment; let's say across gender in equal rates. **R21 Clinical trialist** It’s more difficult to make clinical trials in the academic setting because it’s getting more and more complicated, you have more and more government rules to fulfil, more and more paper you have to fill out. **T8 Principal Investigator of a discontinued trial** The other reason is if it is an indication when there are good other treatment options, so where the clinical study is not the only option where you get for example a very new medication for the disease...that’s also a point where maybe patients are not willing to participate in the study. **R29 Representative of Federal Office of Public Health** The other element of course is, if you make your life difficult by adding protocol requirements that are nice to have rather than really essential, then you slow down your own recruitment because you don’t get the patients that you would need. There is another element that has been used by certain companies that to derail…let’s say development of a competitor product is, you place a very simple study or a relatively simple study in a site and you pay pretty high patient fee, then this might then divert patients to your study rather than into a study from another company. **R22 Representative of pharmaceutical industry**	

Some interviewees highlighted that patients in countries with a “high-quality healthcare system” characterized by availability, accessibility, and affordability of required healthcare through mandatory health insurance often have a number of treatment and care options covered by obligatory health insurance. This influences their willingness to take part in clinical research especially if they do not have an unmet need for treatment and perceive their trial participation to be burdensome. Interviewees further argued that general mistrust of the public in medical research combined with a lack of balanced media coverage creates additional barriers to motivate patients to participate in clinical trials.

Four trialists encountered a change in standard of care for the disease they were investigating due to the new evidence generated from recently completed trials. As a result, they lost the clinical rationale to continue their trial and had to discontinue. A few interviewees elaborated how simultaneously implemented competing trials involving the same kind of participants, sometimes intentionally started by the industry to slow down the trial of the competitor, can lead to poor participant recruitment and early trial discontinuation. Finally, lack of efficient referral of patients between peripheral and tertiary care hospitals for trial recruitment was mentioned as another important hurdle as illustrated in a quote below.

“I think in Scandinavia, it is that patients with special entities which are severe as pancreatitis are only referred to maximal care hospitals. So this is easier. In Germany, this is not the case. Any hospital can treat them and if they see there are too many problems with patients and it’s too bad, they refer them. So there are many patients we do not see at all or referral is too late.” T14, Principal Investigator of a discontinued trial

3. Design-related factors

Most frequently reported design-related factors were the closely related reasons of overoptimistic recruitment estimates and too narrow eligibility criteria (see Table 5 for quotes). Several interviewees reported that an inaccurate initial recruitment assessment formed the basis of trial planning. The following were mentioned as main reasons for inaccurate initial recruitment assessments: (1) a lack of systematic screening of patients within a particular department often due to non-compatible or underutilized electronic medical record systems, (2) an underestimation of the number of patients who would never be approached to participate in a clinical trial (e.g., those visiting the clinic when the study staff was not present, limited awareness about ongoing trials among all clinicians or low motivation among clinicians to recruit patients in the trial), and (3) an underestimation of the number of patients who would refuse participation because “they have no obvious advantage from participating in the study.”

**Table 5:** Quotations illustrating design-related factors

You know, in every study you have the problem that if you look on your emotionally coloured experiences then you mean, ‘Oh, I think from our 2000 patients with disease X, we will have per year a minimum of 100, which can be a part of a trial.’ And then you know, 50% will have no interest to be a member of a clinical trial. ‘Okay we will have 50’. But that’s only possible if everybody in your team has the same motivation as yourself and that’s the problem. And so you see, half of the patients you have no direct contact, despite the situation that I am the chief of the department. And so it’s really a problem of too optimistic view on the clinical basis of your study. **T10 Principal Investigator of a discontinued trial** Everybody knows that the sites will always overestimate their recruitment. But they do it (feasibility assessment) once in the beginning of the study and they base their plans on what the sites say. And then they run into these troubles. And they often don’t repeat the feasibility. If the sites are behind, they are not meeting the targets they said in the beginning, you would ask them again: ‘Ok well you didn’t give us 50 patients, you have 5, can you give us the new plan of what you can actually achieve, now that you have some experience with the study?’ And they would then update their feasibility on an ongoing basis and know exactly where they stand against what is realistic, rather than, you know, wishful thinking. **R6 Representative of pharmaceutical industry** Well, first there is a certain level of optimism, of undue optimism on the part of the researchers. They figure that if these patients come to the hospital; they would be able to recruit them. They don’t necessarily think about what happens at night, during weekends, when the staff maybe goes on vacation, maybe they are not caught when they should be, there will be a good amount of opportunities missed for recruitment. They don’t think about that in advance. And if they do, they underestimate the patients that will be missed during the process. **R25 Representative Clinical Trial Unit** …the second point is wrong assessment by investigators and overestimate of the ability to recruit. My own experience with that – I worked many years in an outpatient department with patients and I participated in quite a large number of studies there were situations where I **completely** (emphasis in original) overestimated my ability to recruit. Oh I thought, yeah no problem I have seen just so many of patients last year here and it happens that from that day onwards there was no patient fulfilling requirements anymore. Just happens! About half a year nothing. **R30 Representative of pharmaceutical industry** The major difficulty usually is the… the exclusion criteria and very tight inclusion criteria. So people are so… let's say, so concerned about having no (…) well, not only confounding factors but also no side effects. So they prevent all patients who could potentially have side effects and they try to exclude them before, which makes it more difficult for us to find exactly the patients… You know, they want to kind of have healthy patients without failure (laughs). Or healthy patients with chronic kidney disease which is relatively rare or healthy diabetics. **T4 Principal Investigator of a discontinued trial** But when you are doing a pilot or feasibility, the sites are handpicked, so these are the keeners, these are not the lagers, it is the people who can recruit and who will recruit and do a good job with it and we feel very strongly that everybody who is eligible for that study should be in that study. We go over and above to make sure that this is going to happen. When we do a pilot, the recruitment rates are estimated but it is the keeners who do that (however that might not be the case of all centers which will be eventually included in the trial). **T1 Research coordinator of a discontinued trial**	

A large number of interviewees, particularly trial investigators, argued that demanding clinical trial protocols and “overzealous researchers who aim to investigate complex research questions within one study often pose many challenges for themselves, trial teams and the participants during trial conduct”; “the more demanding the protocol, the higher the likelihood of protocol violations especially if the trial staff is not adequately trained and supervised.” One interviewee elaborated on this as follows:

“I think there are couple of reasons. One is… who writes the protocols? There is a disconnect between the protocol author and those who need to implement it. Very often you have scientists who write the protocols and they are not familiar enough with the conduct of the clinical trial. Specifically, they get enamored in scientific questions. So let’s add this, let’s add that. I call it in German the ‘Rotkäppchen Syndrom’ [‘Little Red Riding Hood syndrome’]. Where you go to the forest and you walk from mushroom to mushroom and then you get lost in the forest.” R22 Representative of pharmaceutical industry

Representatives of the pharmaceutical industry explained that the desire to participate in an industry-sponsored trial with the prospects of financial incentive for their department compels some clinicians to inflate the number of patients they regularly treat and therefore would be able to recruit in a trial. Other frequently mentioned reasons were the limited number of recruitment sites and study staff, recruitment procedures that were incompatible with routine clinical practice, complex trial design that is difficult to explain to recruiting staff and participants, and trials targeting diseases with low prevalence in the local population.

4. Trial team/recruiter-related factors

Clinical trials require strong engagement, cooperation, and commitment from all the partners and departments involved not only at the beginning but until the end of the study. However, many interviewees had encountered challenging collaborations with colleagues from their own department, other departments within the hospital or other hospitals, which significantly affected their ability to recruit trial participants (see Table 6 for quotes). They also described the perceived hierarchical relationships among various health care professionals as well as ambition and ego of individuals which prevented trial implementation as planned. If the participants were to be referred from other departments, investigators often tried to develop good relationships with their peers, but it was hard to maintain this engagement and commitment until the end of the trial. The motivation of recruiters or the whole trial team also declined over time, if the recruitment period was prolonged several times leading to “trial fatigue.”

**Table 6:** Quotations for trial team/recruiter-related factors

When it comes to including patients and send patients to the pharmacist, this was much more difficult…. mainly the physicians were concerned. And you know that’s all the problems we have: trying to establish collaboration between physicians and pharmacists - is that physicians are always afraid that pharmacists are going to steal their patients and follow their patients instead of them. **T4 Principal Investigator of a discontinued trial** This was a continuous ongoing fight between the chief cardiac surgeon and myself and well… everybody else in the hospital with him. So it was not a funny thing to do, research with him, not really being involved... but well, trying to make our life as researchers difficult. (….) Well, let's put it politely: he was a very strong minded person. To put it a little less polite: he was an extremely difficult redneck. And so what he did, he prohibited… he told a senior doctor that he would no longer support that. What he did, he always said, ‘Well, this patient doesn’t qualify.’ Nobody ever qualified anymore. So the trial was… was going to be dead. **T5 Principal Investigator of a discontinued trial** For rare diseases, it’s really a big challenge. And in general, I mean this non referral - you know, we talk again and again with everybody and they don’t do it (refer). Not even you know, if you think in Zurich, there is hospital A and hospital B. No way would they refer patients to each other! Yeah. I think that this is a big weakness. No large centers and no referral. **R13 Representative of pharmaceutical industry** I think this academic model is outdated. I think it would be much better if we have very skilled clinicians who know how to apply clinical research and do an excellent clinical job and few researchers who first of all do not necessarily have to be MDs, they can also come from other fields, who do really good research and are well trained. And I think it’s certainly correct that we try to recruit these young talented people during different curriculums. (…..) If they are really good then you also have to develop career possibilities and funding for them and that’s also not sufficiently done because, university hospitals want to have good clinicians, good teachers, and good researchers. **R8 Clinical trialist** So it’s not possible to run a trial, an academic trial for instance, with a very interesting research question just by the enthusiasm of all the people involved in all the centers. And this used to be possible about 10 years ago. And now you have so many regulations for the conduct of the trial that you need professional personnel or staff to really conduct it in the centers. There you need money and time. **R20 Clinical trialist**	

Several interviewees believed that the only way academic hospitals could keep up with stringent ethical and regulatory oversight while conducting RCTs is through a professional work force trained in clinical research. Unlike in the past, enthusiasm and motivation of the PI or a few clinicians to undertake research was reported to no longer be sufficient to successfully conduct a RCT with today’s requirements. Interviewees reported that research is often not the main motivation for many clinicians in university hospitals although it is a necessity for career advancement. A representative of a public health authority (R29) expanded on the link between motivation and time investment by the recruiting clinicians as follows:

“The doctors who recruit patients are not necessarily those who sign the contract, who plan the study, who wrote the protocol etc. So maybe this is also why the motivation for recruitment is rather modest and it’s of course time-consuming to recruit patients especially during the busy daily routine, daily practice... It’s the matter of motivation and it’s a matter of time.”

In the current academic set-up, interviewees reported that there are limited incentives to train and retain a professional clinical research workforce due to limited availability of financial resources and career development opportunities. Academic research sites experience high turnover of staff, which influences continuity of proven leadership within a particular trial. Representatives of pharmaceutical industry reflected extensively on this particular factor often juxtaposing it with the industry context.

5. Participant-related factors

Several interviewees reported that the trial participants are reluctant to participate in studies with complex and highly demanding protocols for they perceive it to be burdensome, either in terms of number of visits to the hospital, time to be spent at the recruiting site or regarding procedures to be completed such as collection of biological samples including tissue biopsies, or requirements to withhold standard treatment (that would otherwise already have been started outside a clinical trial) during studies with prolonged follow-up. Some trialists elaborated on this aspect in relation to cancer trials. They argued that patients with advanced stage cancer are likely to be overwhelmed by a RCT, not only physically but also emotionally and are therefore reluctant to participate in trials.

One representative of a pharmaceutical company (R6) was explicit in his reflection about the lack of patient perspective in trial planning and its impact on participant recruitment as illustrated in the quote below.

“One of the root causes (of recruitment failure) I think is that actually almost nobody except the sites actually understand the patients. So to the pharma company the patients are kind of mysterious beings that are something from fairy tales. They never actually have had any interactions with patients. It’s not surprising if they don’t really get the patients perspective because the process in randomized clinical trials, the blinding and everything is keeping such a distance from the patient to sponsor. So how do you design a study for your key stakeholders, for the patients if you never actually seen or spoken with one? So I think lot of it is based on assumptions. Lot of it is based on data coming through the sites, which are often delayed and filtered with the sites perspective.”

A couple of interviewees explained how sometimes a pharmaceutical company intentionally launches a rather simple trial with the same patient population at the same site to slow down the progress of another competing trial with a complicated protocol. In such a situation, both clinicians and patients tend to prefer participation in the simpler and less burdensome trial. Other reported participant-related reasons for recruitment failure were perceived lack of equipoise (uncertainty about the superiority of one intervention over another as a pre-condition for randomization) on the part of participants, e.g., through influence by a next of kin or caring physician, lack of trust due to the shortness of the relationship with the research team, or cultural barriers (see Table 7 for quotes).

**Table 7:** Quotations for participant-related factors

I believe it is a lack of trust between doctor and patient, bad doctor patient relationship, a lack of trust into the system, language barriers, patients not trusting the protocol or not understanding why joining the study should be beneficial for them. It doesn’t always need to be beneficial, there is considerable altruism in patients from my experience, but then altruistic patients want to understand why they are doing study xyz, and if this information is provided, they join. **R24 Representative of patient organization** The main reason I think, sometimes, some studies are too complicated for the patients. Sometimes they are quite full with their cancer history that they have fears and they just… don’t want to focus on other things. In newer studies very often we need a biopsy or repeated biopsies of tumors. It’s difficult to explain that to patients because they don’t have an immediate advantage. I know it’s very interesting and we need data from biopsies, for example for the science. But in the clinic situation, it’s difficult. **T8 Principal Investigator of a discontinued trial** Another important reason is that the protocol - it was a randomized controlled treatment trial in diabetes - the protocol was quite intensive for patients. And a major reason why patients participate or do not participate is whether it brings them something new which they do not receive by conventional, usual daily care. So it was not very attractive. **T9 Principal Investigator of a discontinued trial** …maybe also complicated protocols- require a lot of time from the patient to participate in the study might be another reason why patients if they have another alternative to the treatment do not participate in clinical study. **R29 Representative of Federal Office of Public Health**	

### Comparison with reasons for recruitment failure identified by systematic review

Based on the findings from the present interview study we partly re-structured and extended the previously suggested checklist [4] to the present Table 2. Most of the reasons for recruitment failure mentioned in the interviews corresponded to an existing category of the previous list. However, we identified eight new reasons (in bold typeface) that were mentioned in interviews only (Table 2). In the category “research environment-related reasons”: (1) high-quality health care system with many options limiting patients’ incentive to seek trial participation and (2) few referrals from peripheral hospital to larger (tertiary care) hospitals with research infrastructure. In the category “design-related reasons”: (3) insufficiently compelling research question, (4) too few recruiting sites planned or too few study staff, and (5) lack of patient engagement in trial design/planning. In the category “trial team/recruiter-related reasons”: (6) lack of competence/training/experience of recruiters or the entire trial team and (7) “trial fatigue” due to recurrent prolongation of recruitment. Finally, in the category “participant-related reasons”: (8) lack of trust of patients due to the shortness of the relationship with the trial team.

On the other hand, there were six reasons previously identified in the literature but not mentioned in interviews. In the category “funding-related reasons”: (1) withdrawal of funding when slow recruitment became apparent. In the category “design-related reasons”: (2) unclear eligibility criteria or enrolment process, (3) ineffective screening or advertising strategy, and (4) patients approached in inconvenient situation. Finally, in the category “participant-related reasons”: (5) lack of financial incentive for participants and (6) lack of encouragement from patient support organizations.

Furthermore, we have also now added a new category describing research environment-related factors, i.e., factors related to the health system, research regulation, media, and society. Reasons tapping into this category are typically out of control of trial investigators and therefore hardly preventable. However, prudent researchers check for and consider these factors in their planning and use conservative recruitment estimates in their feasibility deliberations.

## Discussion

This qualitative analysis of 49 semi-structured interviews with clinical trial stakeholders identified a total of 29 different reasons for recruitment failure. Overoptimistic recruitment estimates, too narrow eligibility criteria, lack of engagement of recruiters/trial team, lack of competence/training/experience of recruiters, insufficient initial funding, and high burden for trial participants were mentioned most frequently. This confirms and extends findings of a previous systematic review on published reasons for recruitment failure [4]. Integrating the results of the present study with the complementary systematic review, a checklist of reasons for recruitment failure was revised to now include five main categories: funding-related, research environment-related, design-related, trial team/recruiter-related, and participant-related reasons.

Overall, we believe that most reasons for recruitment failure could have been preventable at the planning and set-up stage through considerations of how the burden for recruiters and participants could be minimized, training and coaching of recruiters to enhance their motivation and knowledge about the trial, and piloting of screening and consent procedures [4, 16, 17]. All listed design-related reasons are basically under the control of investigators. However, available support from clinical trial units or advice from experienced trialists can be instrumental for less experienced investigators to anticipate and master recruitment challenges. Reasons that cannot be controlled or influenced by trial investigators can still be influenced by other stakeholders such as funding agencies, ethics committees, regulatory institutions, or the pharmaceutical industry. These are mainly research environment-related reasons, but some funding-related reasons such as unavailable funds for recruitment prolongation, or withdrawal of funds when slow recruitment becomes apparent, or trial team/recruiter-related reasons such as lack of incentive or lack of competence/experience can be modified by other stakeholders too. Concerted efforts of all involved stakeholders are probably necessary to sustainably reduce recruitment failure in clinical trials.

Research around recruitment to clinical trials is growing [18]. According to the Online resource for Recruitment Research in Clinical triAls (ORCCA) database (www.orrca.org.uk), there are a large number of case reports and studies that evaluate information for participants in clinical trials [18]. However, high-quality evidence on specific interventions aiming to improve participant recruitment is sparse [19–21]. Our study highlights the most frequent causes for recruitment failure and may help guide further methodological empirical research embedded in clinical trials [22, 23]. Several identified root causes for recruitment failure in our study have already been mentioned in the literature as recruitment barriers, e.g., high burden for trial participants, high complexity of the trial protocol, or fear of participants that trial involvement would have a negative effect on the relationship with their physician [9, 10]; but classifying them as root causes for recruitment failure may attribute the necessary significance so that stakeholders act upon those. Consideration should also be given to new trial designs, which suggest increased recruitment efficiency and reduction in trial costs due to shared trial infrastructure and patient screening [24, 25].

Our revised checklist of reasons for recruitment failure may also be useful for researchers, ethics committees, and funding agencies in their assessment of trial feasibility and whether trial investigators have sufficiently anticipated and addressed potential risks of recruitment failure in trial proposals and protocols. The applicability and relevance of each of the listed checklist items can be assessed for an individual RCT in order to identify a specific risk profile that could be used for tailored monitoring during trial conduct and to help estimate an overall risk of recruitment failure at different stages of the trial. Structural challenges and research environment-related factors, however, need to be addressed at institutional, national, or even international levels. All stakeholders basically agreed on the importance of the problem, but it remains less clear to which degree individual stakeholders are prepared to take responsibility and collaborate across stakeholders to tackle the often interlinked bottlenecks.

### Strengths and limitations

Our study included a broad range of clinical trial stakeholders and resulted in new findings complementing the previous systematic research with RCT publications [4]. Previous studies have attempted to identify causes for recruitment failure by statistically testing for associations between candidate variables and recruitment failure. Those analyses, however, typically provide only a superficial view on root causes (mainly due to missing data and lack of power) and are prone to confounding [1, 2, 24]. The qualitative approach taken in this study allowed for a deeper exploration of stakeholders´ experiences with recruitment failure and adds to the scarce evidence base on how recruitment to clinical trials is planned and conducted in practice [7, 8]. Analysis of the interviews was also carried out by a research team to minimize systematic bias that could have arisen if the data had been analyzed by only one researcher. However, there were 61 trial investigators and 4 other stakeholders who did not reply to our emails or declined to participate. Whether the views of those 65 people (57% of invited) are substantially different from those who were interviewed is unclear. Many explained their refusal with their extremely busy schedules and priority given to other tasks; however, it may also be that openly discussing and analyzing failures is not popular in the research community. A second limitation is that we conducted our interviews 5–6 years ago. Although we consider this unlikely, we cannot exclude the possibility that the expressed views by the interviewed stakeholders have changed in the meantime. Third, we only included interviewees from three high-income countries in our study: Switzerland, Germany, and Canada—with 80% (39/49) of interviewees coming from Switzerland. Although we did not find any obvious differences in viewpoints of interviewees depending on the country in our analysis, further research involving more interviewees outside of Switzerland is needed. Fourth, we did not share a preliminary analysis with our interviewees or with those who declined to be interviewed for reassurance that our findings were representative of the data and of others more widely. Fifth, our research team did not include any non-medical health professionals like a nurse or a psychologist. Sixth, with respect to participant-related factors leading to poor recruitment and premature discontinuation of clinical trials we relied on two patient representatives to provide insights in addition to arguments put forth by clinicians and academics. It would have been valuable to additionally include patients who participated in trials discontinued due to poor recruitment in this interview study, which was not feasible. Seventh, we did not go back to the primary data of our systematic review while conducting the root cause analysis of reasons of trial discontinuation in our interview study. So, we cannot exclude the possibility that a newly identified reason might have been present in the primary data of the systematic review but was not recorded that way by the previous research team. Finally, since the publications included in our previous systematic review [4] and the interviewed stakeholders of the present study come from high-income countries, our list of reasons for recruitment failure does not necessarily apply to studies in low and middle-income countries. Further research is needed to examine differences between high-income and low-/middle-income countries with respect to challenges in participant recruitment.

## Conclusions

In conclusion, this study found overoptimistic recruitment estimates, too narrow eligibility criteria, lack of engagement of recruiters/trial team, lack of competence/training/experience of recruiters, insufficient initial funding, and high burden for trial participants to be the most frequently reported reasons for recruitment failure in RCTs. This confirms and extends findings of a previous systematic review on published reasons for recruitment failure and led to an update of a structured checklist summarizing reasons for recruitment failure. The checklist may be useful to guide further research on interventions to improve participant recruitment in RCTs and helpful for trial investigators, research ethics committees, and funding agencies when assessing trial feasibility with respect to recruitment.

## Supplementary Information

**Additional file 1: Appendix A**

**Additional file 2: Appendix B**

## Data Availability

The data used for and/or analyzed during the current study are available upon request from the authors.

## Abbreviations

RCT: Randomized clinical trial
SRQR: Standards for reporting qualitative research
