# Supplementary material for: Exploring reasons for recruitment failure in clinical trials: a qualitative study with clinical trial stakeholders in Switzerland, Germany, and Canada
Source: Trials. 2021 Nov 25;22:844. doi: 10.1186/s13063-021-05818-0 (PMC8613940; doi:10.1186/s13063-021-05818-0)
Supplement: Supplementary file 1 — Additional file 1: Appendix A [file 13063_2021_5818_MOESM1_ESM.doc]

Appendix A

1 - Interview guide - trialists

**Trialist: Interviewer:**

**Trial Ref-ID:**

**SEMI-STRUCTURED TELEPHONE OR IN-PERSON INTERVIEW**

**1. Opening**

Thank trialist for taking part in interview

Give recap of Interview Study (similar to what said in invite email)

Explain phone call will be tape-recorded, transcribed and anonymised

Check enough time for interview (20 minutes approx) (no interruptions / mobiles if possible)

**Questions relating to CV (if not got)**

- Position at time of trial? (and now)
- Professional qualifications at time of trial (MSc or PhD in epidemiology or statistics or clinical research)? (and now)
- Trial experience prior to trial of interest (number of trials as PI, number of trials as co-investigator)? (and now)
- Publication experience prior to trial of interest (number of peer-reviewed original articles)? (and now)

**2. Background to trial:**

- Who funded the trial?
- Who wrote the protocol (PI, co-workers of PI, statistician involved)?

**3. Regarding recruitment:**

- When writing the RCT protocol or when you first read/familiarized yourself with the protocol, did you **anticipate potential problems with recruitment of participants?**
  - **If yes**, what were the anticipated problems, what was planned to prevent/control them, were these considerations documented in the protocol?
  - **If no**, why not?
- When did problems with recruitment become apparent to you?
- What was done to improve recruitment?

**4. Regarding discontinuation:**

- Can you tell me how you decided to discontinue the trial?
- What triggered the discontinuation?
- Who took the decision?
- If interviewee was involved in the decision-making:

a) What were your considerations at the time (any benefits, downsides, difficulties associated with discontinuation)?

b) How did you feel about the trial when the decision was made (relieved?, ashamed?, neither?...) With hindsight, what are the major issues with trial discontinuation for you, if any?

- If ethical aspects were not mentioned: Did you consider any legal or ethical implications at the time?

**5. Process evaluation**

- With hindsight, what were the root-causes for insufficient recruitment in your case?
- Could it have been prevented earlier?
- What are your learned lessons from the experience?
- Any changes on a national level that could improve recruitment to clinical trials?

**6. Dissemination of results after discontinuation**

- Was it important for you to disseminate/publish the results of the discontinued trial?
- Any considerations of benefits, downsides, difficulties associated with publication of results and lessons learned of a discontinued trial?
- In your opinion, what would be the most suitable format to disseminate results from discontinued trials (full journal publication, short report/letter, conference abstract, trial registry, any other website, no publication at all)?

**7. Informing relevant parties about discontinuation**

- Whom did you inform about the discontinuation of the trial?
- Did you consider trial participants and the respective REC? If not, why not?

**8. Close of interview:**

- Do you have any other comments/remarks you would like to make on the topic?

Thank trialist for their contribution, acknowledgement in final report

Copy of results will be sent to them.

2 - Interview guide – other stakeholders

**Stakeholder: Interviewer:**

**SEMI-STRUCTURED TELEPHONE OR IN-PERSON INTERVIEW**

**1. Opening**

Thank stakeholder for taking part in interview

Give recap of Interview Study (similar to what said in invite email)

Explain phone call will be tape-recorded, transcribed and anonymised

Check enough time for interview (20 minutes approx) (no interruptions / mobiles if possible)

**2. Regarding recruitment:**

- What is your experience/perception about the quality/level of detail of information in protocols about participant recruitment?
- In your view, what are the most important reasons/root causes for insufficient recruitment/recruitment problems?
- Can insufficient recruitment be prevented? What could/should be done?
- Any changes on a national level that could improve the situation?

**3. Regarding discontinuation:**

- Coming from poor recruitment to trial discontinuation – if recruitment to a trial is slow, what triggers then in your opinion the actual discontinuation of the trial?
- In your view, what are the major issues with trial discontinuation due to poor recruitment, if any?
- If ethical aspects are not mentioned: Are there any legal or ethical implications of relevance?

**4. Dissemination of information about discontinuation**

- Would you consider it important that inconclusive results of a discontinued trial get published? Why or why not?
- What are the benefits, downsides, difficulties with non-publication of results and lessons learned of a discontinued trial?
- What are potential reasons why many trialists do not inform trial participants and respective RECs about the discontinuation of the trial? How could this be improved?
- In your opinion, what would be the most suitable format to disseminate results from discontinued trials (full journal publication, short report/letter, conference abstract, trial registry, any other website, no publication at all)?

**5. Close of interview:**

- Do you have any other comments/remarks you would like to make on the topic?

Thank stakeholder for their contribution, acknowledgement in final report

Copy of results will be sent to them.
